# A Case of Nontuberculous Mycobacterial Prosthetic Valve Endocarditis

**DOI:** 10.51894/001c.168162

**Published:** 2026-08-28

**Authors:** Siavash Esmaili, Shayan Khan, Carolina Restini, Christopher Hanson, Shaurya Srivastava, David Neff

**Affiliations:** 1 College of Osteopathic Medicine Michigan State University; 2 Heart and Vascular University of Michigan Health Sparrow

**Keywords:** Mycobacterium goodii, Endocarditis, Prosthetic valve infection, ¹⁸F-FDG PET/CT, Nontuberculous mycobacteria, Device-related infections

## Abstract

**INTRODUCTION TO THE TOPIC:**

Prosthetic valve endocarditis (PVE) caused by rapidly growing nontuberculous mycobacteria (NTM) is rare and diagnostically challenging. Cases involving organisms within the Mycobacterium smegmatis group are exceptionally uncommon.

**CASE PRESENTATION:**

We report the case of an 82-year-old man who developed recurrent fevers and persistent Mycobacterium smegmatis bacteremia after bioprosthetic aortic valve replacement and left atrial appendage occlusion. Serial transesophageal echocardiograms remained nondiagnostic despite ongoing symptoms and bacteremia. Subsequent fluorine-18 fluorodeoxyglucose positron emission tomography/computed tomography (¹⁸F-FDG PET/CT) demonstrated intense uptake surrounding the prosthetic aortic valve and the left atrial appendage occlusion device, with maximum standardized uptake values (SUVs) of 9.2 and 5.8, respectively. Repeat surgery revealed a circumferential aortic root abscess, and the valve isolate was reported as Mycobacterium goodii by matrix-assisted laser desorption/ionization time-of-flight mass spectrometry (MALDI-TOF MS). Because both reported identifications fall within the Mycobacterium smegmatis group, the infection was classified at the group level; the species-level assignment remained unresolved. Postoperatively, routine and acid-fast bacillus blood cultures were negative, and the patient’s fevers resolved after surgical source control and antimicrobial therapy. After discharge, the patient completed approximately five months of a clofazimine-containing multidrug antimicrobial regimen. At three years of clinical follow-up, he remained alive, asymptomatic, and free of recurrent infection.

**CONCLUSIONS:**

This case highlights the difficulty of diagnosing NTM prosthetic valve endocarditis when echocardiography is unrevealing and illustrates how PET/CT contributes important diagnostic information in suspected prosthetic cardiac infection. It also adds to the limited literature on Mycobacterium smegmatis group endocarditis and demonstrates the successful incorporation of clofazimine within a multidisciplinary treatment strategy.

## INTRODUCTION

Prosthetic valve endocarditis (PVE) caused by nontuberculous mycobacteria (NTM) is rare, diagnostically challenging, and associated with substantial morbidity and mortality.^1,2^ Among rapidly growing mycobacteria, PVE has been more frequently attributed to organisms such as *Mycobacterium chelonae*, *Mycobacterium fortuitum*, and *Mycobacterium abscessus*, whereas organisms within the *Mycobacterium smegmatis* group have only rarely been implicated.^2,3^ *Mycobacterium smegmatis* sensu stricto, a member of the *Mycobacterium smegmatis* group, is itself an exceptionally uncommon cause of invasive human disease, and the limited historical endocarditis literature largely predates modern species-level classification within the *Mycobacterium smegmatis* group.^4–6^ *Mycobacterium goodii*, also a member of the *Mycobacterium smegmatis* group and first described in 1999, has been associated primarily with post-traumatic wound infections, orthopedic hardware infections, and osteomyelitis; cardiovascular involvement remains exceedingly rare.^6–8^

Published reports of prosthetic valve endocarditis involving organisms within the *Mycobacterium smegmatis* group and closely related rapidly growing mycobacteria are limited (Table 1).^5,7–9^ Two cases were attributed to *Mycobacterium goodii*. Both involved mitral prosthetic material and neurologic septic embolic complications and were managed with surgical replacement and prolonged combination antimicrobial therapy.^7,8^ A third case involved *Mycobacterium wolinskyi* in a patient with multiple cardiac prostheses; routine identification was nondiagnostic, sequencing was required for species identification, and the infection was ultimately controlled with medical therapy alone.^9^ An older postoperative case reported as *Mycobacterium smegmatis* predates modern taxonomic subdivision of the group.^5,6^ The present case is therefore best classified as another prosthetic valve endocarditis case involving the *Mycobacterium smegmatis* group, without a specific ordinal designation. The blood isolates were reported as *Mycobacterium smegmatis*, whereas the explanted valve isolate was reported as *Mycobacterium goodii*. Because both reported identifications fall within the *Mycobacterium smegmatis* group and definitive species-level confirmation was unavailable, the infection is reported at the group level.^6^ Unlike the previously reported *Mycobacterium goodii* cases, the present case involved a bioprosthetic aortic valve with additional left atrial appendage occlusion hardware and remained echocardiographically nondiagnostic despite persistent mycobacterial bacteremia.^7,8^

**Table 1.** Selected published cases of prosthetic valve endocarditis involving organisms within the *Mycobacterium smegmatis* group and closely related rapidly growing mycobacteria, compared with the present case. The table summarizes clinical presentation, diagnostic findings, surgical findings, antimicrobial strategy, and outcomes.

**Publication**	**Pathogen**	**Valve/Device**	**Clinical presentation**	**Key diagnostic findings**	**Surgical findings**	**Antimicrobial strategy**	**Outcome**
**Jönsson G et al., 2012** ^7^	*Mycobacterium goodii*	Prosthetic mitral valve	76-year-old woman; fatigue, fever, confusion, slurred speech, and poor balance; cerebellar ischemic lesion attributed to septic emboli	Blood cultures (positive after approximately 3 days); TEE demonstrated prosthetic valve vegetation, organism identified by 16S rRNA sequencing	Vegetation and infection surrounding the prosthetic valve ring; repeat mitral valve replacement performed	Initial meropenem, gentamicin, and ciprofloxacin; after surgery, ampicillin was followed by tigecycline plus ciprofloxacin, then oral doxycycline plus ciprofloxacin at discharge; total treatment approximately 4 months	Cured; no relapse at 30-week follow-up
**Parikh RB et al., 2017** ^8^	*Mycobacterium goodii*	Mitral valve annuloplasty ring	67-year-old man; fever, loss of appetite, gait disturbance 3 weeks after mitral ring annuloplasty; brain MRI showed multiple ring-enhancing lesions suggestive of septic emboli	Blood cultures (initially suggested *Actinomyces*); TEE demonstrated vegetation and annuloplasty-ring involvement; 16S rRNA sequencing matched *Mycobacterium goodii* (100%), although *Mycobacterium smegmatis* could not be excluded (99.5% homology)	Mitral valve replacement; intraoperative vegetations confirmed	Empiric multidrug therapy, then ciprofloxacin plus trimethoprim-sulfamethoxazole for total 6 months after susceptibility testing	Cured; symptom-free at 137-week follow-up
**Kitajima H et al., 2021** ^9^	*Mycobacterium wolinskyi*	Aortic and mitral valve replacements and left atrial appendage closure	82-year-old man; recurrent syncope and fever after cardiac surgery; right temporal hemorrhagic infarction	Blood cultures positive after 90 hours; MALDI-TOF MS suggested *Mycobacterium wolinskyi* but did not reach a definitive score; identification confirmed by 16S rRNA, *hsp65*, and *rpoB* sequencing	Managed without surgery	Amikacin, imipenem, and clarithromycin for 6 weeks, followed by oral ciprofloxacin and minocycline to complete 12 months of therapy	Cured; infection controlled without surgery
**Present case**	Blood isolates reported as *Mycobacterium smegmatis*; valve isolate reported as *Mycobacterium goodii*; final classification: *Mycobacterium smegmatis* group	Bioprosthetic aortic valve and AtriClip/left atrial appendage occlusion hardware	82-year-old man; recurrent fevers and mycobacterial bacteremia after aortic valve replacement and AtriClip placement	Serial TEE nondiagnostic; PET/CT showed uptake at the aortic valve prosthesis (SUV 9.2) and AtriClip (SUV 5.8); valve isolate reported as *Mycobacterium goodii* by MALDI-TOF MS in 2023	Redo sternotomy; gross prosthetic valve infection; circumferential aortic root abscess; AtriClip removed	Multidrug regimens; susceptibility-guided preoperative linezolid, moxifloxacin, and minocycline; postoperative regimen modified and included clofazimine	Cured; alive and asymptomatic at follow-up in May 2026

hsp65, heat shock protein 65 gene; LAA, left atrial appendage; MALDI-TOF MS, matrix-assisted laser desorption/ionization time-of-flight mass spectrometry; MRI, magnetic resonance imaging; PET/CT, positron emission tomography/computed tomography; rpoB, RNA polymerase β-subunit gene; rRNA, ribosomal RNA; SUV, standardized uptake value; TEE, transesophageal echocardiography.

Nontuberculous mycobacterial prosthetic valve endocarditis may present subacutely with nonspecific symptoms and persistent or intermittent bacteremia.^10^ Standard echocardiographic evaluation may repeatedly be nondiagnostic, particularly when infection involves prosthetic material or periannular structures.^11,12^ Advanced imaging is therefore often used when clinical suspicion for PVE remains despite negative or inconclusive echocardiography.^11,12^ Current diagnostic frameworks support this approach: the 2023 European Society of Cardiology (ESC) guidelines recommend fluorine-18 fluorodeoxyglucose positron emission tomography/computed tomography (^18^F-FDG PET/CT) in patients with suspected PVE when echocardiography is inconclusive, and the 2023 Duke–International Society for Cardiovascular Infectious Diseases (ISCVID) criteria incorporate abnormal ^18^F-FDG PET/CT metabolic activity involving a prosthetic valve, intracardiac device leads, or other prosthetic material as a major imaging criterion when PET/CT is performed at least 3 months after implantation of the prosthetic material.^11,13^ In this context, PET/CT can help localize metabolically active prosthetic infection, identify extracardiac foci, and guide decisions regarding surgical source control.^11,12^

In parallel with evolving diagnostic strategies, therapeutic options for rapidly growing NTM infections remain limited by antimicrobial resistance patterns and medication intolerance, particularly when prosthetic material is involved.^10,14^ Clofazimine, historically used in leprosy, has been used as an adjunct in multidrug regimens for NTM infections based on in vitro activity and limited clinical experience; however, evidence supporting its use in NTM endocarditis remains very limited, and its use in this setting is off-label.^15–17^ We report a case of *Mycobacterium smegmatis* group prosthetic aortic valve endocarditis with dual prosthetic involvement, in which serial transesophageal echocardiography was nondiagnostic and PET/CT contributed important diagnostic information that supported surgical source control.

An 82-year-old man with severe aortic stenosis underwent bioprosthetic aortic valve replacement with a 25-mm Magna Ease valve and left atrial appendage occlusion using an AtriClip device in April 2021. His relevant medical history included hypertension, hyperlipidemia, hypothyroidism, stage 2 chronic kidney disease, hiatal hernia, iron-deficiency anemia, prior squamous cell carcinoma, and remote tobacco use. He had no documented history of diabetes mellitus, immunosuppressive therapy, or prior mycobacterial bacteremia. Baseline outpatient medications included amlodipine 10 mg once daily and levothyroxine 25 µg once daily, along with vitamin and iron supplementation. Before the onset of illness, the patient lived at home with his wife, was ambulatory without assistance, and was independent in activities of daily living. Following surgery, his cardiovascular status improved, but he did not return to his previous functional baseline. He subsequently developed recurrent fevers beginning in late July or early August 2021.

At that stage, the differential diagnosis remained broad and included occult postoperative infection, noncardiac infectious sources, noninfectious causes of fever, and prosthetic valve endocarditis. A timeline of the major diagnostic and therapeutic events is presented in Table 2.

**Table 2.** Clinical timeline of major diagnostic and therapeutic events.

Date	Clinical event or finding
04/08/2021	**Bioprosthetic aortic valve replacement** 25-mm Magna Ease valve with LAA occlusion using an AtriClip device
Late July–early August 2021	**Onset of symptoms** Recurrent fevers of unknown origin
12/15/2021	**Brain MRI: acute ischemia** Tiny focus in the inferior right cerebellum without hemorrhage
12/20/2021	**First positive blood culture** *Mycobacterium smegmatis*
December 2021	**TEE** Abnormally thickened prosthetic leaflets without definitive vegetations
December 2021	**Initial antimicrobial therapy** intravenous meropenem + oral doxycycline + oral ethambutol for 6 weeks
Early 2022 to April 2022	**Transition to outpatient therapy** Continued on an oral antimicrobial regimen; therapy was briefly discontinued around April 2022
April 2022	**Recurrent symptoms after treatment interruption** Resumed doxycycline, clarithromycin, and ethambutol; doxycycline was later replaced with TMP-SMX
09/30/2022	**Repeat TEE: well-seated valve, nondiagnostic** No vegetations or paravalvular leak
03/30/2023	**PET/CT: intense dual uptake** Aortic valve prosthesis, SUV 9.2; AtriClip device, SUV 5.8
05/09/2023	**Redo sternotomy with aortic root debridement** Circumferential aortic root abscess; valve isolate subsequently reported as *Mycobacterium goodii* (2023)
05/30/2023	**Leadless pacemaker (Micra AV) implanted** For postoperative complete AV block
06/22/2023	**Discharged on clofazimine-containing multidrug antimicrobial regimen** Continued for approximately five months after discharge
02/13/2024	**Cardiac MRI follow-up** LVEF 57%, moderate MR, no pericardial constriction

Abbreviations: AV, atrioventricular; LAA, left atrial appendage; LVEF, left ventricular ejection fraction; MR, mitral regurgitation; MRI, magnetic resonance imaging; PET/CT, positron emission tomography/computed tomography; SUV, standardized uptake value; TEE, transesophageal echocardiography; TMP-SMX, trimethoprim-sulfamethoxazole.

In December 2021, blood cultures were intermittently positive for *Mycobacterium smegmatis*, with positive cultures interspersed with negative routine and acid-fast bacillus (AFB) blood cultures through April 2023 (Table 3).

**Table 3.** Serial blood cultures with intermittent positivity.

Date	Specimen	Result	Organism
12/20/2021	Blood culture (routine)	**+**	*Mycobacterium smegmatis*
12/26/2021	Blood culture (routine)	**–**	No growth
12/30/2021	Blood culture (routine)	**+**	*Mycobacterium smegmatis*
12/30/2021	AFB blood culture	**–**	No growth
04/06/2022	AFB blood culture	**–**	No growth
05/02/2022	Blood culture (routine)	**+**	*Mycobacterium smegmatis*
06/13/2022	Blood culture (routine)	**+**	*Mycobacterium smegmatis*
10/17/2022	AFB blood culture	**–**	No growth
01/24/2023	AFB blood culture	**–**	No growth
02/01/2023	Blood culture (routine)	**–**	No growth
02/10/2023	Blood culture (routine)	**+**	*Mycobacterium smegmatis*
04/24/2023	Blood culture (outside hospital)	**+**	*Mycobacterium smegmatis*

*Result symbols: (+) positive; (–) no growth.* AFB, acid-fast bacillus. The 04/24/2023 culture was the last positive culture before the redo valve replacement on 05/09/2023; no sustained microbiologic clearance was documented prior to surgery. *Cultures never durably cleared before the redo valve replacement on 05/09/2023.*

**Figure 1.**
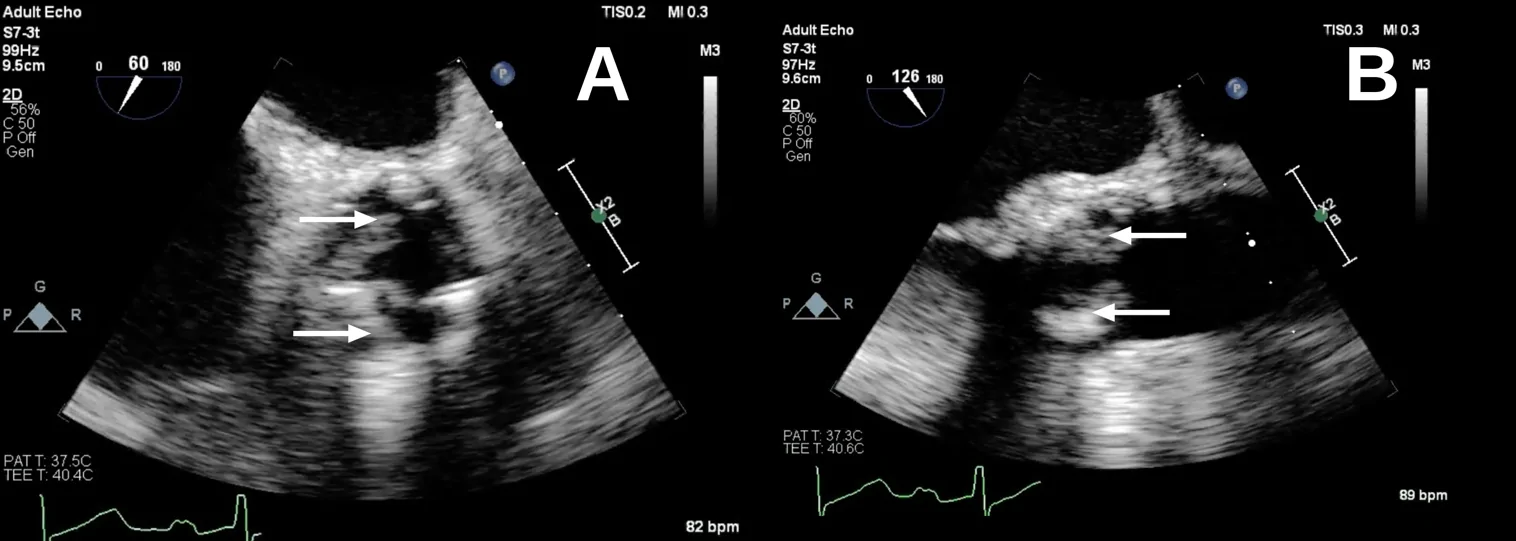
Transesophageal echocardiography in December 2021. (A) View obtained at a multiplane angle of 60°; (B) view obtained at 126°. Arrows indicate abnormally thickened bioprosthetic aortic valve leaflets without definitive vegetations or periannular complications.

Concurrent transesophageal echocardiography (TEE) performed in December 2021 demonstrated abnormally thickened prosthetic valve leaflets but no definitive vegetations or periannular complications (Figure 1).

Taken together, these findings did not establish a definitive diagnosis of PVE. Rapidly growing NTM are uncommon causes of endovascular infection.^10,14^ The bacteremia was intermittent, and TEE did not demonstrate vegetations or periannular complications. At that time, contamination, transient bacteremia, and noncardiac sources of fever remained plausible considerations. Accordingly, immediate reoperation was not pursued. Instead, the patient was managed with antimicrobial therapy and close clinical and microbiological surveillance. Initial therapy consisted of a 6-week course of intravenous meropenem, oral doxycycline, and oral ethambutol, followed by an outpatient oral regimen, as summarized in Table 2.

During this period, brain magnetic resonance imaging (MRI), obtained in December 2021 for evaluation of headache, demonstrated a tiny acute ischemic infarct in the inferior right cerebellum without hemorrhage. At that time, the lesion was considered an incidental finding and was not attributed to septic embolization because PVE had not yet been established.

Antimicrobial therapy was briefly discontinued around April 2022, but symptoms recurred within several weeks, prompting resumption of doxycycline, clarithromycin, and ethambutol. Doxycycline was subsequently replaced with trimethoprim-sulfamethoxazole (TMP-SMX), although the exact durations of these outpatient regimens were not consistently documented. Despite ongoing treatment, blood cultures remained intermittently positive.

A repeat TEE in September 2022 demonstrated a well-seated, normally functioning bioprosthetic aortic valve without vegetations, preserved biventricular systolic function, and no intracardiac thrombus. Given the absence of definitive echocardiographic evidence of PVE, the patient continued to be managed with antimicrobial therapy, surveillance blood cultures, and close follow-up rather than undergoing immediate reoperation. However, recurrent fevers and intermittent mycobacterial bacteremia (Table 3) persisted despite multiple antimicrobial regimens, making contamination or transient bacteremia less likely and increasing concern for occult prosthetic valve or device-related infection not adequately visualized by echocardiography.

Antimicrobial susceptibility testing of the blood isolate, performed in February 2023, showed low minimum inhibitory concentrations (MICs) for several oral agents, including linezolid, moxifloxacin, minocycline, and clofazimine (Table 4).

**Table 4.** Antimicrobial susceptibilities by isolate.

Antibiotic (MIC, µg/mL)	Explanted valve isolate reported as *Mycobacterium goodii* in 2023*	*Mycobacterium smegmatis* group blood isolate**
Amikacin	**≤1 (S)**	**8 (S)**
Amoxicillin/clavulanate	—	8/4
Azithromycin	—	64
Cefepime	—	>64
Cefoxitin	**>128 (R)**	—
Ceftriaxone	—	128
Ciprofloxacin	**S**	<1
Clarithromycin	**>16 (R)**	2
Clofazimine	0.25	<0.5
Doxycycline	**0.25 (S)**	<1
Imipenem	8	—
Linezolid	**8 (S)**	<1
Minocycline	—	<0.5
Moxifloxacin	0.25	<0.5
Tigecycline	0.25	**S**
Tobramycin	**8 (R)**	—
TMP-SMX	**2/38 (S)**	**S**

MIC, minimum inhibitory concentration; S, susceptible; R, resistant; “—”, agent not tested on that panel. The blood-isolate report provided MICs without formal interpretation except where shown. The valve-isolate report noted that tobramycin breakpoints apply only to Mycobacterium chelonae and that clarithromycin is the macrolide. * Susceptibility testing performed by the Mayo Clinic (July 3, 2023). ** Susceptibility testing performed by the National Jewish Health laboratory (February 10, 2023).

**Figure 2.**
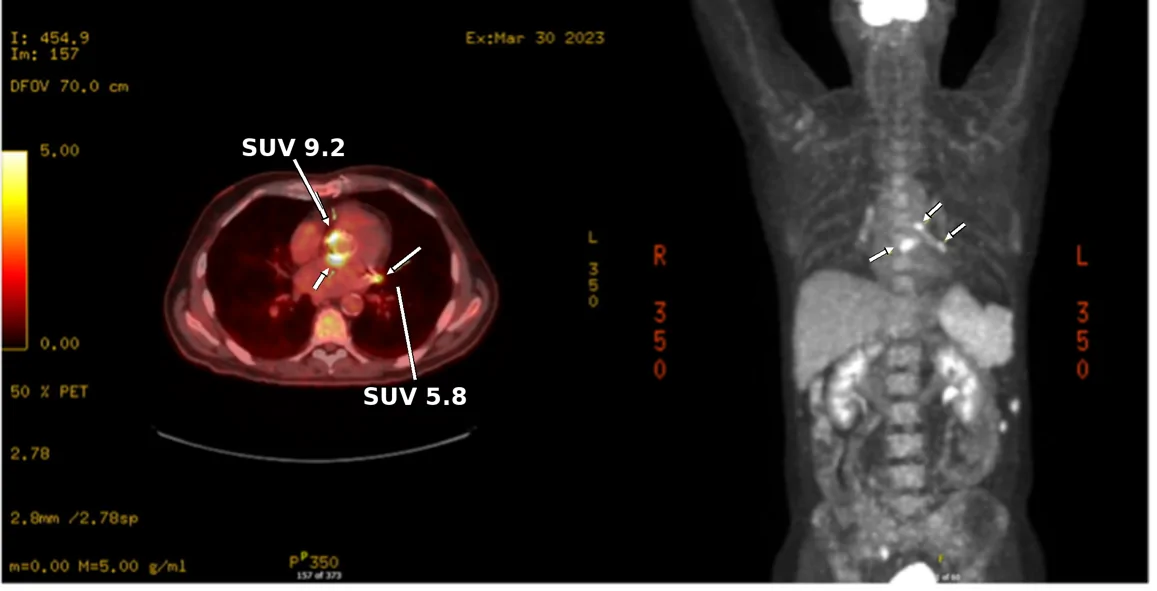
Fluorine-18 fluorodeoxyglucose positron emission tomography/computed tomography (^18^F-FDG PET/CT) in March 2023. Arrows indicate areas of intense metabolic uptake involving the bioprosthetic aortic valve and left atrial appendage occlusion hardware.

Persistent bacteremia and recurrent fevers prompted advanced imaging with PET/CT in March 2023, which demonstrated intense uptake surrounding the aortic valve prosthesis (maximum standardized uptake value [SUV] of 9.2) and focal uptake at both ends of the AtriClip device (SUV of 5.8), raising concern for infectious or inflammatory involvement of prosthetic material (Figure 2).

Because recurrent fevers and mycobacterial bacteremia persisted despite the preceding multidrug regimen, the infectious diseases team changed therapy in April 2023 to linezolid 600 mg once daily, moxifloxacin 400 mg once daily, and minocycline 100 mg twice daily. This regimen was consistent with the February 2023 susceptibility profile, which demonstrated low MICs for linezolid (<1 µg/mL), moxifloxacin (<0.5 µg/mL), and minocycline (<0.5 µg/mL).

Given persistent mycobacterial bacteremia and PET/CT findings localizing suspected involvement of the aortic valve prosthesis and left atrial appendage occlusion hardware, the anticipated benefits of repeat valve surgery were judged to outweigh the considerable operative risks, including those associated with the patient’s advanced age.

On May 9, 2023, the patient underwent redo sternotomy with explantation of the infected bioprosthetic aortic valve, aggressive aortic root debridement and circumferential reconstruction using a bovine pericardial patch, and implantation of a 25-mm bioprosthetic valve. The AtriClip device was removed, and the left atrial appendage was oversewn.

Intraoperative findings demonstrated a grossly infected prosthetic valve with thickened and discolored leaflets and numerous small vegetations. A circumferential abscess cavity was present within the aortic root, with destruction of the entire aortic annulus, the aorto-mitral continuity, and most of the left ventricular outflow tract, necessitating aggressive debridement and circumferential bovine pericardial reconstruction. Operative specimens were sent for routine bacterial, anaerobic, fungal, and acid-fast bacillus (AFB) cultures. Routine bacterial, anaerobic, and fungal cultures showed no growth. AFB culture of the explanted valve grew a rapidly growing mycobacterium. At that time, the valve isolate was reported as *Mycobacterium goodii* based on matrix-assisted laser desorption/ionization time-of-flight mass spectrometry (MALDI-TOF). Because *Mycobacterium goodii* is a member of the *Mycobacterium smegmatis* group,^6^ this identification was consistent at the group level with the patient’s earlier blood isolates reported as *Mycobacterium smegmatis*. Surgical pathology of soft tissue from the explanted AtriClip device demonstrated a foreign body giant cell reaction.

The postoperative course was complicated by complete atrioventricular block with pacing dependence, requiring implantation of a Micra AV leadless permanent pacemaker in May 2023. The postoperative regimen required modification because of lactic acidosis, thrombocytopenia, and hepatotoxicity. Oral clofazimine (100 mg once daily) was incorporated into the prolonged multidrug therapy because susceptibility testing demonstrated a low MIC and other standard oral options were limited by toxicity or poor tolerability. The patient was discharged in June 2023 on a clofazimine-containing multidrug antimicrobial regimen, which was continued for approximately five months after hospital discharge.

Follow-up routine and acid-fast bacillus (AFB) blood cultures, obtained from May 10 through September 13, 2023, showed no growth (Table 5). No subsequent microbiologic evidence of recurrence was documented. Fever resolved following surgical source control and completion of antimicrobial therapy.

**Table 5.** Postoperative surveillance blood cultures.

**Date**	**Specimen**	**Result**	**Note**
05/10/2023	Blood culture (routine)	**–**	No growth
05/10/2023	AFB blood culture	**–**	No growth
05/16/2023	Blood culture (routine) ×2	**–**	No growth
05/17/2023	Blood culture (routine)	**–**	No growth
05/19/2023	AFB blood culture	**–**	No growth
09/13/2023	Blood culture (routine)	**–**	No growth
09/13/2023	AFB blood culture	**–**	No growth

AFB, acid-fast bacillus; -, negative result. Following redo aortic valve replacement and root abscess repair on 05/09/2023 (the explanted valve isolate was reported as Mycobacterium goodii in 2023), all surveillance cultures were negative.

A transthoracic echocardiogram showed eccentric mitral regurgitation that was difficult to quantify. Cardiac magnetic resonance imaging performed in February 2024 demonstrated moderate mitral regurgitation, a preserved left ventricular ejection fraction of 57%, and no evidence of prosthetic aortic valve dysfunction.

At the most recent follow-up in May 2026, approximately three years after redo aortic valve surgery, the patient remained asymptomatic with no clinical evidence of recurrent infection. Since discharge, he has experienced no infection-related rehospitalizations, has returned to his baseline functional status, and has remained independent in activities of daily living. He continued routine follow-up with cardiology and primary care.

## PATIENT PERSPECTIVE

From the patient’s perspective, the prolonged diagnostic course was characterized primarily by fatigue and reduced physical reserve during recurrent febrile episodes. Following definitive treatment, he reported substantial improvement in quality of life and a return to baseline functional status.

## DISCUSSION

This case highlights the diagnostic challenges of PVE caused by rapidly growing NTM in the setting of persistent or relapsing bacteremia and repeatedly nondiagnostic echocardiography. Although TEE remains central to the evaluation of suspected PVE, its sensitivity may be limited when infection involves prosthetic material, periannular tissue, or early abscess formation without a discrete vegetation.^11,12^ In the present case, serial TEE demonstrated only subtle prosthetic leaflet thickening without definitive evidence of infective endocarditis, whereas operative findings revealed extensive prosthetic valve infection and a circumferential aortic root abscess. This discordance represents a key feature of this case and reinforces that negative or equivocal echocardiographic findings should not be used to exclude prosthetic valve infection when persistent mycobacterial bacteremia is present.

The present case shared several features with the *Mycobacterium goodii* prosthetic valve endocarditis cases reported by Jönsson et al. and Parikh and Grant: a subacute presentation, involvement of prosthetic cardiac material, possible neurologic embolization, and the need for prolonged multidrug antimicrobial therapy and surgical source control.^7,8^ Given the limited literature on prosthetic valve endocarditis involving the *Mycobacterium smegmatis* group and closely related rapidly growing NTM, this case adds to the literature by illustrating an atypical presentation characterized by equivocal TEE findings and intermittent bacteremia.^5,7–9^ In retrospect, the cerebellar infarct may have represented an embolic phenomenon associated with the subsequently confirmed prosthetic valve and device infection, although a definitive embolic source was not established in the present case.

In contrast, both prior cases involved mitral prosthetic material, whereas the present case involved a bioprosthetic aortic valve with additional left atrial appendage occlusion hardware. Unlike the previously reported *Mycobacterium goodii* prosthetic valve cases, in which TEE demonstrated vegetations or other definitive prosthetic involvement, serial TEE in the present case remained nondiagnostic despite persistent mycobacterial bacteremia.^7,8^ PET/CT localized suspected prosthetic infection, which was ultimately confirmed surgically.

In this case, PET/CT contributed important diagnostic information by localizing metabolically active abnormalities involving both the aortic valve prosthesis and left atrial appendage occlusion hardware. These findings, interpreted alongside persistent mycobacterial bacteremia and recurrent fevers, supported the decision to pursue surgical source control. Although this single case cannot establish diagnostic superiority of PET/CT over echocardiography, it illustrates how PET/CT may complement structural imaging when clinical suspicion for prosthetic valve or device-related infection remains high despite nondiagnostic TEE.^11,12^ This case also aligns with contemporary diagnostic frameworks, including the 2023 Duke–ISCVID criteria, which recognize abnormal metabolic activity involving a prosthetic valve or intracardiac device as a major imaging criterion when PET/CT is performed at least three months after prosthetic valve implantation.^13^

Antimicrobial management of rapidly growing NTM infections involving prosthetic material is often constrained by limited clinical experience, prolonged treatment courses, and medication intolerance.^10,14^ Clofazimine, historically used to treat leprosy, has been used off-label as part of multidrug regimens for difficult-to-treat NTM infections, with support from in vitro activity and limited clinical experience.^15–17^ However, evidence supporting clofazimine in NTM endocarditis remains extremely limited,^10^ and its independent contribution in this case cannot be determined because it was used alongside surgical source control and other antimicrobial agents. In the present case, clofazimine was incorporated as an adjunctive component of prolonged multidrug therapy when standard oral options were limited by adverse effects and susceptibility concerns.

## LIMITATIONS

Several limitations should be considered. This report describes a single patient with an exceptionally rare prosthetic valve infection, and the findings should therefore be interpreted cautiously.

The microbiologic findings should be interpreted in chronological context. The blood isolates were reported as *Mycobacterium smegmatis*, whereas the explanted valve isolate was identified in 2023 as *Mycobacterium goodii* by MALDI-TOF. Because *Mycobacterium goodii* is a member of the *Mycobacterium smegmatis* group,^6^ the findings were concordant at the group level but discordant at the species level. The Mayo Clinic susceptibility report used the identification supplied by the submitting laboratory and therefore did not independently resolve this discrepancy. In the absence of independent confirmatory testing, the infection is best classified within the *Mycobacterium smegmatis* group, while the species-level assignment remains unresolved.

This single case report cannot establish the diagnostic superiority of PET/CT or the efficacy of clofazimine in NTM prosthetic valve endocarditis. Because clofazimine was used as part of a broader strategy that included surgical source control and concomitant antimicrobial therapy, its independent contribution cannot be determined.

## CONCLUSION

This case adds to the limited literature on prosthetic valve endocarditis involving organisms within the *Mycobacterium smegmatis* group.^5,7–9^ Although the blood and valve isolates were reported as *Mycobacterium smegmatis* and *Mycobacterium goodii*, respectively, the species-level identification remained inconclusive. Persistent NTM bacteremia may reflect prosthetic infection even when serial echocardiography is unrevealing.^10,12^ In this case, PET/CT contributed important diagnostic information by localizing suspected involvement of both the prosthetic aortic valve and left atrial appendage occlusion hardware. These findings prompted surgical source control, and operative examination confirmed a circumferential aortic root abscess. This case highlights the rarity and diagnostic challenges of NTM prosthetic valve endocarditis and illustrates the potential value of multimodality imaging and multidisciplinary management in complex prosthetic cardiac infections.^11,12^ Therapeutic conclusions regarding the adjunctive use of clofazimine remain limited by the single-patient nature of this report.

### Conflict of Interest

The authors declare no conflicts of interest.

### Consent to Publish

Written informed consent for publication of this de-identified case report was obtained from the patient.
